# I53-50: Engineered icosahedral protein cage for modular vaccine nanoplatform

**DOI:** 10.71150/jm.2511020

**Published:** 2026-04-06

**Authors:** Ke Liang, Shuang Wu, Sihang Dong, Tao Xu, Hongtao Wang

**Affiliations:** Anhui Province Key Laboratory of Immunology in Chronic Diseases, Laboratory Medicine Experimental Center, Laboratory Medicine College, Bengbu Medical University, Bengbu 233030, P. R. China

**Keywords:** I53-50, nanoparticles, self-assembly, vaccine, gene fusion

## Abstract

I53-50 is a computationally designed, self-assembling protein nanoparticle (NP) that forms a stable icosahedral structure composed of 120 protein subunits coordinated through precise interfacial interactions. Through unique intelligent regulation, I53-50 exhibits sensitivity to environmental signals and display multimodal “nano-smart” properties. I53-50 has a variety of modifiable surface-active sites, which facilitates precise chemical modification, gene fusion, tag coupling, and other functionalizations, thereby promoting effective lymphatic uptake and optimizing the immune response. I53-50 NPs show great potential in vaccine development, drug delivery, and biomaterials, representing a model fusion of computational biology and nanomedicine and offering a versatile tool for precision medicine.

I53-50 nanoparticles (I53-50 NPs) are protein-based, self-assembling nanostructures artificially designed and developed through a collaborative effort involving the University of Washington, the Howard Hughes Medical Institute, and the University of California, Los Angeles in 2016 (Bale et al., 2016). Their design leverages the self-assembling mechanisms of natural protein NPs. The core architecture of I53-50 originates from the integration of computational biology and structural protein engineering. Unlike natural NP-forming systems such as viral capsids or ferritin-based assemblies, I53-50 is entirely synthetic and therefore offers programmable control over size, porosity, and surface chemistry, effectively circumventing the immunogenicity limitations of natural vectors. I53-50 is composed of trimeric I53-50A and pentameric I53-50B, whose separate expression and purification followed by mixing leads to efficient self-assembly into highly ordered and homogeneous icosahedral particles. Remarkably, the in vitro assembly rate of these independently purified components is comparable to the rapid kinetics observed in viral capsid formation. I53-50A and I53-50B are arranged along the 5- and 3-fold icosahedral symmetry axes, respectively. This high degree of symmetry enables their assembly in the presence of cargo molecules, allowing efficient packaging. Controlled cargo encapsulation can be successfully achieved through charge complementation. Therefore, I53-50 provides a valuable platform for research across diverse fields, including vaccine development, targeted drug delivery, and disease diagnosis and treatment.

In this review, we focus on the structural properties of I53-50 NPs, their functionalization, and their applications in vaccine development and other biomedical domains, as to provide a reference framework for subsequent research exploring their design, optimization, and translational potential.

## Structure and Properties of I53-50 NPs

### Structural features

I53-50 NPs are complexes of multiple artificially designed protein subunits that undergo in vitro self-assembly via non-covalent interactions. These subunits are computationally designed to optimize their interaction interfaces to ensure the spontaneous formation of stable nanoscale structures under specific conditions. The I53-50 NP has a molecular weight of approximately 2.5 MDa and a diameter ranging from 26 to 31 nm. This size is comparable to that of a small viral capsid, and the structure contains more than 130,000 heavy atoms. The I53-50 NP is formed by the in vitro self-assembly of two oligomeric protein components: the trimeric I53-50A and the pentameric I53-50B. I53-50A (20 copies) is used to genetically fuse the target antigens; I53-50B (12 copies) serves as the scaffold core for the NP assembly. As a hierarchical structure, I53-50 relies on artificially designed protein monomers at its base. The sequences of these monomers, which usually contain 200 to 300 amino acid residues, are precisely optimized by computational software. The interaction interface is crucial to the design process, as it ensures the monomers can self-assemble in a predetermined manner (Janin et al., 2008). These monomer sequences often contain specific structural domain modalities such as α-helices and β-folds, secondary structural elements that provide the structural basis for advanced assembly. At the secondary structure level, each monomer contains precisely designed structural modules. The structural domain at the N-terminal end of a typical I53-50 monomer is primarily responsible for coordinating interactions with other monomers; the core domain provides structural stability, and the C-terminal domain participates in interface recognition and assembly regulation (Brodin et al., 2012). These domains are connected by flexible polypeptide linkers that enable the conformational rearrangements for successful self-assembly. The tertiary structure of I53-50 is characterized by the spatial folding of individual protein monomers. As shown by X-ray crystallography and cryo-electron microscopy, these monomers fold into a compact globular structure with specific interacting interfaces distributed on their surfaces. These interfaces have been precisely calculated to ensure their spontaneous assembly under appropriate conditions (Hura et al., 2009). The quaternary structure of I53-50 comprises 120 protein subunits derived from two non-identical components (I53-50A and I53-50B) that self-assemble into a nanocage with T = 4 icosahedral symmetry via non-covalent interactions. This architecture is the most distinctive feature of I53-50 and provides the structural basis for its function. The asymmetric unit is a heterodimer, consisting of one subunit from each of the two components (Grueninger et al., 2008).

## Properties of I53-50

### Structural stability

I53-50 NPs exhibit excellent structural integrity due to their artificially designed unique structure, featuring a rigid icosahedral framework with a Young’s modulus of up to 1.2 GPa. Dynamic light scattering analysis shows that the particle size distribution index (PDI) consistently remains below 0.1 in the temperature range of 4–60°C, maintaining structural integrity for more than 72 h (Bale et al., 2015). Furthermore, the melting temperature (Tm), determined by differential scanning calorimetry (DSC), is 75 ± 2°C, a value significantly higher than that of most natural protein NPs. I53-50 shows strong resistance to a wide range of physiologically relevant stressors, tolerating urea concentrations up to 500 mM; remaining stable in 1% SDS solution for up to 6 h (Padilla et al., 2001); and being 3–5 times more resistant to degradation by common proteases (e.g., trypsin) than natural proteins. This stability stems from its precisely designed, tightly packed structure and optimized subunit interfacial interactions, making I53-50 an ideal candidate for nanobiotechnology applications.

### Intelligent regulation

**Fig. 2.**
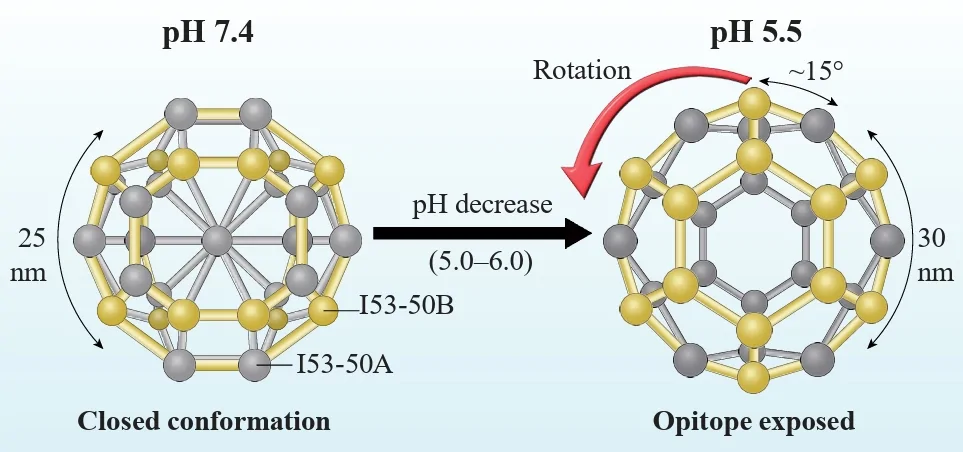
PH‑triggered conformational change of the I53‑50 Nanoparticles.

The most significant technical advantage of I53-50 NPs lies in their programmable intelligent regulation, enabling dynamic sensing, signal integration, and autonomous response to environmental signals through biomimetic engineering. This grants the NPs multi-modal “nano-intelligent” characteristics, which facilitates precise and controllable drug delivery and treatment. Embedding a pH-responsive switching domain within the I53-50 protein subunit through targeted mutagenesis allows the I53-50 NP to maintain exceptional stability at neutral systemic pH 7.4 while enabling rapid disassembly and cargo release upon reaching the mildly acidic tumor microenvironment (pH 6.5–6.8) or endosomes, thereby facilitating precise, stimuli-triggered delivery. At the molecular level, this trigger operates as the pH change leads to the protonation of histidine residues, initiating a structural shift in the components of I53-50 that triggers a 15–45° subunit rotation (Fig. 2). This rotation simultaneously exposes hidden membrane-penetrating peptide sequences and induces the release of encapsulated drug molecules. The resulting selective release, coupled with the NP’s stability in normal tissues, significantly reduces the systemic toxicity of conventional chemotherapy (Tang et al., 2024). Moreover, the entire process is completely reversible: the particles automatically regain their original conformation upon returning to neutral pH, exhibiting a “nanobot”-like self-resetting behavior. In addition, I53-50 is endowed with programmable biomolecular control through artificial coding, which enhances targeting specificity and enables site-specific release. This allows a single NP system to integrate multiple functions, such as environmental sensing, target accumulation, and stimulus-responsive drug release, exemplifying the core attributes of “smart nanomedicine” (Shen et al., 2022). I53-50 NPs embody a level of intelligent regulation that is difficult to achieve with traditional materials, enabled by molecular-scale sensing, processing, and actuation components. Their innovation lies not only in executing single-response functions, but in the seamless integration of multiple intelligent modules into a nanoscale system capable of autonomous decision-making.

### Self-assembly properties

The I53-50 NPs are hollow spherical particles with a diameter of about 26–31 nm and an internal cavity of 15–20 nm, suitable for encapsulating functional molecules. The computationally designed protein self-assembly system underpins the structural synergy and dynamic regulation that define I53-50 NPs as functional nanomaterials (Yao et al., 2009). The synergistic nature of self-assembly is manifested by the rapid completion of structural growth after initial nucleation, resulting in NPs with uniform size and stable structure. Environmental factors such as pH and ion concentration can modulate the assembly process, reflecting the condition-dependent dynamic equilibrium properties of I53-50. The self-assembly of I53-50 is governed by the molecular-level programming of its trimeric and pentameric subunits, relying on non-covalent interaction forces, such as electrostatic complementarity and hydrophobic interactions, as well as C-terminal regions that contribute to stability and assembly fidelity (Lawrence and Colman, 1993). This modality enables I53-50 to serve not only as a structural scaffold but also lays the foundation for subsequent functional expansion. By integrating antigenic epitopes or engineering specific surface sites within the subunit sequences, viral or bacterial antigens can be displayed. This dual self-organization of structure and function makes I53-50 NPs an ideal platform for vaccine development. Among functionally extended self-assembly systems, I53-50 stands out for its robust multivalent display capacity (Goodsell and Olson, 2000). For example, researchers fused the receptor-binding domain (RBD) antigen of SARS-CoV-2 to the N-terminal end of the pentameric I53-50B subunit via a 15-amino-acid flexible linker (Brouwer et al., 2019). This modification preserved the subunit’s self-assembly capacity and enabled the ordered display of 60 RBD antigens on the surface of each NP. Animal results showed that this NP-based vaccine elicited neutralizing antibody (nAb) titers approximately 20-fold higher than those induced by conventional monomeric vaccines and stimulated stronger T-cell immune responses (Pancera et al., 2014). The programmability of the self-assembly process allowed researchers to tailor the physicochemical properties of I53-50 by adjusting subunit sequence parameters, fully demonstrating the unique advantages of I53-50 NPs for precise structural control.

### Functionalization of I53-50 nanoparticles

Functionalization refers to the process of modifying the surface of NPs by chemical or physical methods to introduce specific functional groups or molecules, thereby altering their properties and functions. I53-50 NPs can be modified through chemical conjugation, gene fusion, label coupling, and other strategies—such as surface and cavity engineering—to further optimize their dispersion, stability, biocompatibility, targeting specificity, and responsiveness (Table 1).

### Chemical modification

Chemical modification of I53-50 involves targeting intrinsic active residues to attach functional groups or molecules, enabling protein conjugation and overcoming spatial constraints for enhanced functionality. I53-50 presents a surface with 120 active subunits, each offering multiple sites that are readily modifiable. This high number of accessible sites translates to vast potential for customization and functionalization. The structural features most similar to those of I53-50 are found in the natural architecture of cowpea mosaic virus (CPMV), which possesses a 120-subunit capsid with pseudo-T = 3 symmetry (Lin et al., 1999). I53-50’s N-terminal and C-terminal regions are the most common sites for chemical modification, allowing functional molecules to be displayed directly on the outer surface and facilitating binding to macromolecules.

For example (Brouwer et al., 2019), in the chimeric BG505 SOSIP.v5.2-I53-50A NP construct, which displays the human immunodeficiency virus type 1 (HIV-1) envelope glycoprotein (Env) trimer (SOSIP) on the I53-50A subunit, the distance between the BG505 SOSIP.v5.2 trimer and the I53-50A subunit is 16 Å (van Gils et al., 2016). Considering the heuristic of 1–2 amino acids for every 2 Å of linear distance, a linker of 12 amino acid residues was selected for modification. Specifically, the EKAAKAEEAARK linker was used to replace the first two amino acids (MK) in the N-terminus of the I53-50A subunit. This modification serves a dual purpose: it extends the I53-50A subunit’s N-terminal helix toward the NP’s outer surface, thereby precisely covering the 16 Å distance between the two binding domains, while simultaneously consolidating the overall stability of the chimeric construct. Crucially, the use of this cloning procedure and connector facilitated the generation of I53-50A.1NT1, I53-50A.1NT2, and I53-50A.1PT1 variants, which greatly improved the efficiency of vaccine production.

Using genetic fusion (recombinant DNA technology), a series of I53-50-based fusion proteins were designed to enhance the immunological potency of HIV viral antibodies (Brinkkemper et al., 2024). Brouwer et al. (2021) successfully presented 20 stable SOSIP trimers from different strains on the self-assembling NPs. Stabilization was primarily achieved by introducing an artificial disulfide bond (SOS) linking the gp120 and gp41 subunits at the trimerization site, along with an isoleucine-to-proline mutation (I559P) in the extracellular domain of gp41. These modifications improved antibody response quality, especially in SOSIP trimers presenting apex-proximal neutralizing epitopes, compared to conventional single-component NP systems.

### Tag coupling

Tag conjugation of I53-50 NPs involves functionalizing protein subunits by introducing specific tags—via genetic engineering or chemical methods—for purification, detection, and targeting. These approaches enable site-specific, covalent coupling and have the advantages of being highly stable and fast reacting. The 13-amino acid SpyTag (ST) and the 15 kDa SpyCatcher (SC), developed in 2012, are split-protein components derived from the fibronectin-binding protein FbaB of *Streptococcus pyogenes*. They spontaneously form irreversible covalent isopeptide bonds upon recognition (Buldun et al., 2018). This efficient covalent protein coupling tool enables site-specific binding under mild conditions, and integration of SpyTag or SpyCatcher into I53-50 nanoparticles allows modular functional modifications (Rahikainen et al., 2021). The irreversible linkage formed between SpyTag and SpyCatcher allows the protein NPs to bind to antigenic molecules (Eom et al., 2024). Kang et al. (2021) utilized a covalent linkage strategy and selected the RBD of the SARS-CoV-2 Spike protein (S protein) as an ideal target for vaccine development, constructing three different RBD-coupled NP vaccine candidates. For the I53-50 NP candidate, the ΔN1-SpyCatcher was first genetically fused to one component of the I53-50 NP, with the resulting scaffold then being covalently coupled to a SpyTag attached to the C-terminal end of the RBD via the spontaneous SpyTag/SpyCatcher reaction (Keeble et al., 2019). This coupling is based on the formation of a highly stable, irreversible isopeptide bond, which enables the high-density display and quantitative conjugation of antigens on the surface of I53-50 for subsequent immune presentation. The physical stability of I53-50 NPs was further assessed by nanodifferential scanning fluorimetry (nanoDSF), revealing that RBD-I53-50 NPs aggregate at approximately 70°C, a temperature near the intrinsic thermal stability of monomeric I53-50 and markedly higher than that of the RBD monomer. Therefore, the conjugate vaccine maintains significantly higher stability than the RBD monomer even at conventional vaccine storage temperatures of 4°C, making the production process highly favorable for commercial production and distribution.

### Gene fusion

Gene fusion is the most common and direct method used for antigen presentation in NPs, where the coded antigenic gene is fused to a modified NP gene carrier. This enables protein self-assembly and gene delivery to converge, facilitating optimal antigenic display on the nanoparticle. I53-50 NPs are formed by the self-assembly of 120 subunits, whose gene sequences can be precisely modified without disrupting overall assembly. Each subunit contains a stabilized rigid framework region and a modifiable linker region, typically incorporating either a flexible (G_4_S)_n_ linker or a rigid EAAAK linker to enhance antigen presentation flexibility (Hsia et al., 2021). Rigid frameworks maintain icosahedral symmetry structures, whereas flexible linker sequences located in the N-terminal, C-terminal, or surface loop regions, such as the loop region of I53-50B, allow the insertion of short peptide antigens (Bale et al., 2015). It was shown that insertion of peptides up to 50 amino acids long at the C-terminal end of the subunit or sequences of 30 amino acids at the N-terminal end did not significantly affect the self-assembly efficiency of the particles. This structural tolerance provides an ideal molecular basis for gene fusion in vaccine development.

Genetically fusing the gene encoding the viral antigenic determinant directly to the gene of an I53-50 subunit protein enables high-density antigenic epitope display. In this way, between 60 and 240 antigenic copies can be displayed per assembled NP (~50 nm particle). For example, Marcandalli’s team (Marcandalli et al., 2019) presented the stabilized trimeric respiratory syncytial virus F (RSV-F) glycoprotein immunogen, DS-Cav1, on the surface of I53-50 NPs. In mouse and non-human primate studies, the fully-valent NP immunogen, displaying approximately 20 DS-Cav1 trimers, induced a nAb response 10-fold greater than that induced by the trimeric DS-Cav1 antigen alone. Moreover, recent research indicates that gene fusion technology can be primarily used to link the RSV preF antigen (first-generation DS-Cav1, second-generation Sc9-10) to the trimeric I53-50A subunit sequence (Hu et al., 2025). Additionally, a C-terminal T4 fibronectin trimerization motif (Foldon) was introduced into the preF sequence to facilitate trimer formation. The Sc9-10 antigen fusion sequence also contains a furin cleavage site mutation to prevent cleavage during protein maturation. The codon-optimized fusion gene was transfected into sTable

Chinese hamster ovary (CHO) cells to express the “preF-Foldon-I53-50A” fusion protein in a secreted form. Subsequently, it self-assembled with the I53-50B subunit, which was expressed and purified from the *E. coli* BL21(DE3) expression system, to form the Sc9-10-I53-50 NP. This design significantly enhances antigen stability and immunogenicity.

Jiang et al. (2025) similarly employed direct gene fusion technology to display the DS2 antigen. Following immunization of BALB/c mice with the DS2-I53-50 NP vaccine, this conjugate demonstrated significant advantages over vaccines using free DS2 antigen or ferritin and lumazine synthase as carriers. Regarding humoral immunity, the vaccine induced DS2-specific IgG titers 2.9 times higher than free DS2, with the IgG1/IgG2a ratio reduced to 2.6, indicating a shift toward a protective Th1-type response. It also produced optimal nAb titers against RSV prototype strains (LONG, 18537) and circulating strains (ON1, BA9), with nAb titers against the LONG strain being 2.4 times higher than those induced by free DS2. Regarding cellular immunity, it significantly promoted germinal center B cell and follicular helper T cell proliferation, activated dendritic cells and macrophages, and expanded CD4⁺ central/effector memory T cell populations. Regarding challenge protection, mice exhibited the fastest weight recovery post-challenge, with lung viral load reduced by 3.7 log compared to the PBS group. Lung pathological damage scores were only 1.4, showing minimal alveolar septal thickening and inflammatory infiltration. Overall, the DS2-I53-50 NP vaccine demonstrated the most optimal profile with respect to both immune protection efficacy and safety.

Pascha et al. (2024) genetically engineered the multimerization component (CompA) in I53-50A to undergo a C-terminal fusion with the meningococcal factor H-binding protein (fHbp) antigen, specifically His-labeled peptide 55, via a 12-residue Gly-Ser (GS) junction (fHbp-CompA). This fusion protein co-assembled with pentameric I53-50B to form an I53-50 NP which ultimately displayed 60 copies of peptide 55 on its surface. The Hbp-NP demonstrated significantly more efficient triggering of functional antibody responses to closely related fHbp variants than other NP platforms, indicating a broader and more potent immune activation. The enhanced levels of bactericidal activity ranged from 15-fold to 16-fold higher relative to the licensed meningococcal vaccine MenB-FHbp (Trumenba) and more than 200-fold higher relative to low-valent antigens (i.e., the soluble, monomeric, or dimeric forms of the fHbp antigen alone). Moreover, only mice immunized with fHbp-NP exhibited levels of bactericidal activity that exceeded the established protective relevance against MenB isolates expressing distantly related fHbp variants. These results suggest that immunization with fHbp-NP elicits a more effective antibody response with greater coverage than an equivalent amount of peptide 55 delivered as a low-valent antigen or MenB-FHbp.

Interestingly, the two-component I53-50 NP platform computationally designed by Brinkkemper’s team (Brinkkemper et al., 2024) demonstrates the capacity for mosaic display of multiple distinct Spike (S) proteins, suggesting a novel strategy for addressing SARS-Cov-2 variability (Brouwer et al., 2021). Since the primary targets of SARS-CoV-2 nAbs are structural domains on the viral S protein—specifically the RBDs and N-terminal domains (NTDs)—next-generation vaccine design must account for these regions as they appear in emerging, mutated SARS-CoV-2 variants. To broaden antigenic coverage, the team employed a GS junction to genetically fuse the N-terminal end of CompA to the C-terminal end of the proline-stabilized, pre-fused Beta S protein to expand the range of sarbecovirus S proteins displayed on the NP, thus enhancing the potential for broad sarbecovirus immunization.

**Fig. 3.**
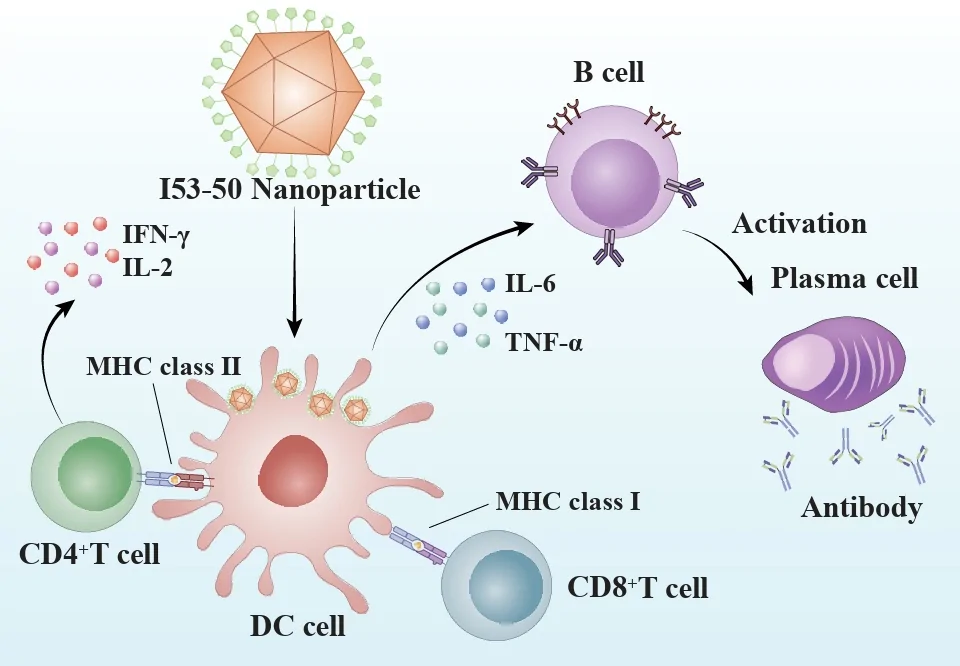
Schematic illustration of the I53-50 Nanoparticles-mediated immune activation.

In summary, through rational structural design and precise functional modifications, the I53-50 nanometer platform achieves highly efficient immune activation. To clearly illustrate this integrated strategy and its cascading biological effects, Fig. 3 outlines its core mechanism of action (Fig. 3). To substantiate this integrated strategy with quantifiable and comparative evidence, Table 2 presents a systematic, side‑by‑side analysis of critical design parameters—including subunit stoichiometry, thermal stability (Tm), and antigen display valency—relative to other widely used protein scaffolds. Together, these elements provide a coherent, evidence‑based rationale for the selection of I53‑50 as a robust and configurable platform for rational vaccine design (Table 2).

## Biomedical Applications of I53-50 NPs

### Vaccine development

I53-50 NPS show significant advantages for viral vaccine development due to their high programmability and structural stability. Through rational design and optimization, they enable the creation of highly efficient vaccine candidates targeting a broad spectrum of pathogens. This technology has been successfully applied in the development of vaccines for influenza, HIV, RSV, and SARS-CoV-2, demonstrating good application prospects (Table 3).

### HIV vaccines

Env is a key surface protein of HIV-1 that facilitates viral entry by mediating receptor binding and membrane fusion with host cells. Env is the principal target for nAbs and serves as a key candidate antigen in the design of HIV vaccines. A major challenge in HIV vaccine research is the rapid mutability of the virus, and the general consensus in the field is that an effective vaccine will likely need to elicit a broad neutralizing antibody (bNAb) response to overcome the sequence diversity of the Env protein (Jia et al., 2015). To date, scientists have developed stable versions of Env such as the SOSIP trimer, which have been shown to elicit nAb responses in vivo; however, these responses tend to be strain-specific, limiting their breadth across diverse viral variants. To induce bNAb responses, researchers—including Brinkkemper’s team (Brinkkemper et al., 2024)—have made notable progress through the design and development of novel HIV-1 mRNA vaccines. This strategy leverages the ability to display stabilized SOSIP Env trimers from multiple HIV-1 strains on I53-50 NPs. The stability of the SOSIP trimer is achieved by synthetic engineering, including an optimized cleavage site between gp41 and gp120, an intermolecular SOS bond linking gp120 and gp41 to prevent dissociation, and a proline mutation in the gp41 ectodomain that locks the trimer in its pre-fusion state (Brouwer and Sanders, 2019). Since I53-50 nanoparticles can be produced in a quality-controlled manner with SOSIP Env trimers prior to assembly, the two-component NP can also promote co-presentation of different antigens to facilitate interactions with cross-reactive B-cells, resulting in improved protection against influenza and hepatitis C viruses and heterologous nAb responses in animal models (Boyoglu-Barnum et al., 2021). The I53-50 NP platform improves the quality of the immune response compared to conventional single-component NP vaccines. Specifically, the delivery of SOSIP on I53-50 improves the antigen-antibody binding response by more than 10-fold. The Env antigen’s success in triggering the nAb response to apical-proximal neutralizing epitopes may benefit even more from the structured presentation offered by the I53-50 NPs (Kanekiyo et al., 2019).

### Epstein-Barr virus

gB, a key glycoprotein in the envelope of the Epstein-Barr virus (EBV), plays a central role in viral entry and cell-to-cell transmission, and is currently the leading antigen in EBV vaccine development. Due to their large surface area and optimal size for lymphatic drainage, I53-50 NPs can display multiple copies of gB, thereby enhancing antigen capture and immune recognition. Sun et al. (2023) calculated the optimal linkage spacing between the gB antigen and the I53-50 NP components and evaluated their structural compatibility using a coaxial docking algorithm, which informed the design of the gB-I53-50 vaccine. To this end, the I53-50B.4PT1 pentameric subunit was expressed and purified from an *E. coli* expression system. The final NPs were then formed in vitro through the noncovalent interaction between purified I53-50B.4PT1 and the gB antigen fused to the I53-50A1 trimeric component, resulting in gB protruding symmetrically on the surface of the NP.

Compared with traditional subunit vaccines, the gB-I53-50 NP vaccine displays multiple copies of gB on its surface, enhancing antigen exposure and density to effectively improve immunogenicity in vivo. In addition, the vaccine efficiently activates cytotoxic T cell responses, enabling the targeted elimination of virus-infected host cells and further strengthening antiviral immunity. Serum polyclonal antibodies induced in monkeys by the gB-I53-50 NP vaccine could be passively transferred and significantly protect humanized mice from EBV infection and lymphoma development. Crucially, the protective effect of the antibodies remained potent 10 weeks after the monkeys’ immunization, demonstrating that the vaccine elicits robust and durable nAb responses.

### SARS-CoV-2 vaccines

During the development of a novel vaccine against SARS-CoV-2, Brouwer’s research team (Brouwer et al., 2021) designed and synthesized the I53-50 NP platform specifically to serve as a potent delivery vehicle for the viral S protein. The construct was designed to create a fusion protein by genetically linking the C-terminus of the prefusion S protein to the N-terminus of the I53-50A component variant I53-50A.1NT1. The resulting S-I53-50A.1NT1 fusion protein was then expressed and purified from human embryonic kidney (HEK) 293F cells (Bai et al., 2021). The final SARS-CoV-2 S-I53-50 NP, formed in vitro after incubation with the I53-50B.4PT1 subunit, was used to immunize rhesus macaques. The study’s final results demonstrated several key protective effects, including a stable nAb response, reduced lung damage, decreased lymphocyte depletion, and a significant reduction in viral load and replication through both the upper and lower respiratory tracts. The strong preclinical data for the S-I53-50 NP in generating durable nAbs and protection in macaques strongly supports the viability of this design approach for future human vaccines that can both reduce the risk of severe SARS-CoV-2 pathologies and effectively control virus shedding and transmission.

Notably, in 2022, an I53-50-based COVID-19 vaccine, marketed as SKYCovione (GBP510) was approved for use in South Korea (Song et al., 2022). The vaccine displays the SARS-CoV-2 RBD on the I53-50 NP scaffold and is adjuvanted with AS03. This design was used to present multiple (likely 60) RBD antigens on the I53-50 NP for optimal immune presentation. Three different vaccine constructs, RBD-8G-I53-50, RBD-12G-I53-50, and RBD-16G-I53-50, were created by attaching the RBDs to the I53-50 NP scaffold via flexible protein linkers composed, respectively, of 8, 12, and 16 GS residues. The NP vaccine constructs induced extremely high nAb titers—with only a low dose—that were up to 10-fold higher than those induced by monomeric S protein (Arunachalam et al., 2021). This remarkable potency demonstrates that protein NP vaccines, such as those built on the I53-50 platform, possess higher immunogenicity and can induce efficient immune effects with a low antigen dose. This finding, which directly promoted the advancement of the SARS-CoV-2 S-RBD NP vaccine into the clinical stage, provides a strong rationale for the development of I53-50 NPs as a superior vaccine carrier.

### Hepatitis C virus vaccines

Two glycoproteins, E1 and E2, are embedded in the outer envelope of the hepatitis C virus (HCV) and represent the sole targets for eliciting nAbs, making them key components in HCV vaccine development (He et al., 2020). Most recombinant HCV glycoprotein vaccines elicit only weak nAb responses, as they typically include only the E2 glycoprotein. To overcome this limitation, Sliepen et al. (2022) engineered a recombinant soluble E2E1 trimer by arranging the E1 and E2 glycoproteins, and conjugated it to the I53-50A component via the C-terminus of E1 to form the E2E1-I53-50A fusion protein. E2E1-I53-50A was then assembled with I53-50B.4PT1 to derive the final E2E1-I53-50 NP via size exclusion chromatography. In vivo studies showed that the number of nAbs generated in vivo (ID_50_ > 40) was significantly higher in animals immunized with E2E1-I53-50 NPs than in those receiving E2 monomer immunization. Furthermore, the breadth and potency of the nAb response were significantly improved, with an increase in potency of up to 80-fold. These findings show that the E2E1-I53-50 NP vaccine induces high nAb titers, possesses enhanced epitope affinity for bNAbs, and elicits a nAb response capable of targeting a wider range of viral strains. This superior immunological profile validates a promising strategy for designing future HCV vaccines.

### East Coast fever vaccine

Lacasta et al. (2023) addressed the fatal impact of East Coast fever (ECF), a tick-borne disease affecting cattle, by engineering I53-50 NPs to display 60 copies of p67C, a poorly immunogenic surface protein from the *Theileria parva* sporozoite stage. The researchers linked p67C to the I53-50A N-terminus, and the resulting p67C-I53-50A fusion gene and I53-50B.4PT1 were individually cloned into the pET29b^+^ vector. These plasmids were expressed in *E. coli* strains (Lemo21(DE3) or BL21 Star (DE3)). The final p67C-I53-50 NP was then formed in vitro by mixing the purified p67C-I53-50A trimers with the purified I53-50B.4PT1 pentamers. In animal immunization studies, p67C-I53-50 NPs elicited the strongest p67C-specific IgG1 and IgG2 responses, robust CD4^+^ T cell activation, and effective sporozoite neutralization. On day 70 post-immunization, enriched CD4^+^ T cells were assayed for proliferative IFN-γ secretion upon antigen stimulation. The p67C-I53-50 NP outperformed soluble antigen s-p67C and two other NPs tested, p67C-I32-28 and p67C-I32-19. Moreover, p67C-I53-50 induced a more favorable antibody response than HBcAg-p67C virus-like particles (VLPs).

### I53-50 NPs in tumor immunotherapy

In recent years, the I53-50 NP system has achieved breakthroughs in tumor immunotherapy, with its unique structural and immunomodulatory properties offering a novel platform for cancer treatment (Bhardwaj et al., 2020). Studies have shown that NP technology significantly enhances anti-tumor immune responses through multidimensional mechanisms and holds great promise for both basic research and clinical translation (Yang et al., 2011). The distinctive internal cavity and highly engineered surface of I53-50 make it an attractive modular platform for potential applications in tumor immunotherapy. First, its highly ordered and repetitive protein surface structure resembles that of natural VLPs. This feature has been widely shown to be efficiently recognized by antigen-presenting cells, such as dendritic cells, and to provide “danger signals” through the activation of pattern recognition receptors (e.g., Toll-like receptors), thereby conferring a potential self-adjuvant effect (Gou et al., 2024). Tumor-specific antigens or neoantigens can be precisely displayed on the surface of the I53-50 NP by gene fusion or chemical coupling; at the same time, its lumen can encapsulate small-molecule immune adjuvants. This “antigen-adjuvant” co-delivery strategy mimics the pathogen model, theoretically enhancing the immunogenicity of the antigen and promoting a potent and specific T-cell response (Lee et al., 2025). Second, its structurally stable internal cavity enables the delivery of immunomodulatory molecules, such as easily degradable cytokines or immune checkpoint-blocking antibody fragments, facilitating their protection and targeted release within the tumor microenvironment to enhance therapeutic efficacy and reduce systemic toxicity (Mao et al., 2024).

### I53-50 NPs in drug delivery

I53-50 NPs exhibit unique advantages and strong application potential in drug delivery systems. Their highly programmable icosahedral architecture and precise atomic-level assembly provide an ideal platform for drug loading and targeted delivery, opening new avenues for therapeutic research (Kreuter, 1996). By genetically engineering the intraluminal surface of the I53-50 NP, a hollow structure with a diameter of about 16 nm can be constructed, which provides a natural loading space for hydrophobic small molecule chemotherapeutic drugs, imaging probes, nucleic acid-based drugs (e.g., siRNA, miRNA) or protein/peptide drugs (Lai et al., 2014). The inherent stability of the I53-50 protein shell allows it to encapsulate the payload and effectively protect the cargo drug from enzymatic degradation in the bloodstream, immune clearance, and premature release, significantly enhancing its in vivo stability and half-life. The multifunctional design of the NP system supports highly efficient molecular targeting and offers formulation flexibility through the integration of active targeting elements and functionalized visualization agents.

## Discussion

In recent years, I53-50 NPs have demonstrated strong application potential across several biomedical fields. Ferritin requires intracellular assembly, which can result in antigen misfolding and non-nAb responses, while VLPs are complex to produce and typically exhibit low antigen loading density. I53-50 NPs adopt a sophisticated, distinctive two-component assembly architecture that enhances structural complexity and engineering controllability, thereby enabling the precise spatial presentation of multiple antigens. In contrast to Mi3 nanoparticles, I53-50 NPs possess larger morphological dimensions, which endow them with expanded surface areas and enlarged internal cavities to accommodate elevated copy numbers of antigens with superior loading capacity.

The precise structural architecture of the I53-50 NPs platform confers its potent immunogenicity by enabling specific immunological mechanisms. The ordered, high-density antigen arrays on its surface maximize B-cell receptor (BCR) cross-linking, delivering a potent initial activation signal that drives the generation of high-titer neutralizing antibodies (Bachmann and Jennings, 2010). Furthermore, the platform stably presents antigens in their native conformations, creating a persistent antigen reservoir that continuously promotes antibody affinity maturation and breadth development within germinal centers (Reddy et al., 2007). Complementing this, the nanoscale size of the particles facilitates their efficient uptake by antigen-presenting cells (APCs), leading to the activation of antigen-specific CD4^+^ T-helper cells, which provide critical support for B-cell responses and immune memory formation (Maia et al., 2024). Consequently, by orchestrating multiple synergistic steps—from antigen presentation and lymphocyte activation to memory generation—the platform achieves integrated and potent humoral and cellular immunity.

Chemical modification of I53-50 enables the covalent attachment of active moieties or targeted biomolecular groups to its outer surface. This strategy offers flexible reaction conditions, broad applicability, and the capacity to incorporate non-natural functional molecules. Gene fusion enables artificial targeting modification of I53-50, while chemical approaches, supplemented by biological methods, allow site-specific functionalization. By tailoring the modification strategy to specific needs, I53-50 can achieve a broader functional repertoire, positioning it as a nanocarrier with promising development prospects in biomedical applications. Since I53-50 NPs exhibit excellent stability, certain formulations remain active at room temperature for extended periods, which is crucial for vaccine storage and transportation. In addition, vaccines based on I53-50 NPs offer enhanced safety, rapid production timelines, and scalability for mass manufacturing. Their ability to incorporate multiple antigenic epitopes on a single I53-50 surface facilitates the development of broad-spectrum vaccines and may challenge existing vaccine paradigms by enabling more modular and standardized preparation strategies (Delehanty et al., 2010).

Currently, vaccine formulations based on I53-50 NPs have progressed to the development stage, with a few already reaching the market through large-scale production. In contrast, research on I53-50 NPs for drug delivery remains in its early stages and has yet to advance to clinical trials.

Although significant progress has been made in the development of vaccines based on I53-50 NPs, several challenges and potential risks remain that must be addressed. First, spatial and conformational constraints limit the ability of individual subunits on I53-50 NPs to couple efficiently with target proteins or peptides, often requiring sequence optimization. Moreover, the requirement for two distinct components to achieve NP assembly adds complexity to the production process. These factors have hindered the development of I53-50 NPs in vaccine design. Therefore, reducing the complexity of assembly while enhancing the safety of the assembled nanoparticles will improve their applicability as carriers for vaccine delivery. The approved SKYCovione (GBP510) vaccine provides a systematic solution model. By introducing stabilizing mutations such as Rpk9 into the RBD antigen, it significantly enhances the intrinsic thermal stability of the protein component, thereby improving the robustness of subsequent assembly processes against process fluctuations. Simultaneously, its computationally designed I53-50 platform interface, leveraging highly complementary strong electrostatic interactions, endows the assembly process with intrinsic self-correction and self-driving capabilities. This effectively reduces stringent requirements for feed stoichiometry precision while ensuring product homogeneity. More importantly, its platform-based production strategy—which employs a division of labor between mammalian and prokaryotic systems for expression and integrates in vitro assembly as a standardized unit operation into the process flow—successfully achieved stable scale-up from laboratory scale to GMP-compliant commercial production. This case study demonstrates that proactively integrating rational upstream protein design with scalable downstream platform processes is key to overcoming manufacturing challenges for complex biologics and bridging the gap from innovative design to affordable product commercialization.

In addition, it remains unclear whether modifications may alter the antigenic structure or compromise the structural stability of I53-50 NPs. Priority should be given to feasible strategies for mitigating scaffold immunogenicity, including surface masking (e.g., coating with biomacromolecules such as hyaluronic acid, albumin, or even natural cell membranes) and PEGylation (i.e., covalent conjugation of polyethylene glycol) (Becicka et al., 2021). Specifically, surface masking using natural cell membranes has been shown to enable nanocarriers to evade immune recognition and clearance by mimicking the immune compatibility of endogenous cells, thereby significantly reducing their immunogenicity and extending in vivo circulation time. Meanwhile, PEGylation can form a hydrated shell on the surface of nanocarriers to shield immunogenic epitopes, which is a well-established strategy for reducing the immunogenicity of synthetic nanoplatforms in both preclinical and clinical settings (Almalik et al., 2017). Furthermore, the use of I53-50 as an antigen carrier carries a potential risk of eliciting antibody response against the NP scaffold itself (Ramos-Pan et al., 2024). While highly versatile, SpyCatcher/SpyTag proteins are exogenous proteins that may be immunogenic in vivo. Most I53-50 NP vectors are constructed using genetic engineering, resulting in more complex preparation and purification processes and higher production costs compared to traditional platforms. Therefore, most I53-50 NP-based formulations remain in the basic research stage. It is noteworthy that the traditional computational design paradigm, exemplified by the I53-50 system, is being transformed by next-generation AI tools. Models such as RFdiffusion and ProteinMPNN enable a function-oriented design approach. RFdiffusion can generate novel protein scaffolds with desired symmetries or pores, while ProteinMPNN efficiently designs stable sequences for these backbones and optimizes interfacial interactions (Dauparas et al., 2022; Frank et al., 2024). This synergy expands the design space, allowing for the creation of tailored I53-50 variants with multiplexed functionalities, thereby advancing their applications beyond vaccines toward smart delivery and synthetic biology. There is also a need to establish a better safety evaluation system for nanomaterials, as to address the challenge of real-time monitoring of I53-50 NPs in complex biological environments, as well as to reduce the cost of production to improve their accessibility. An in-depth understanding of the interaction mechanisms between I53-50 and biological systems, especially the potential risks that may be brought by long-term exposure, will be a critical focus of future research. Accelerating the transition from basic research to clinical translation will be essential for advancing the safe and effective medical application of I53-50 NPs.

As the aforementioned challenges are gradually resolved, the effective design of more dynamically controllable I53-50 nanoparticles will be enabled. This advancement is expected to broaden the development prospects of I53-50 research and accelerate progress toward a new generation of nanoformulations with impactful applications in biomedical fields, including disease diagnosis, prevention, and treatment.

## Figures and Tables

**Fig. 1.**
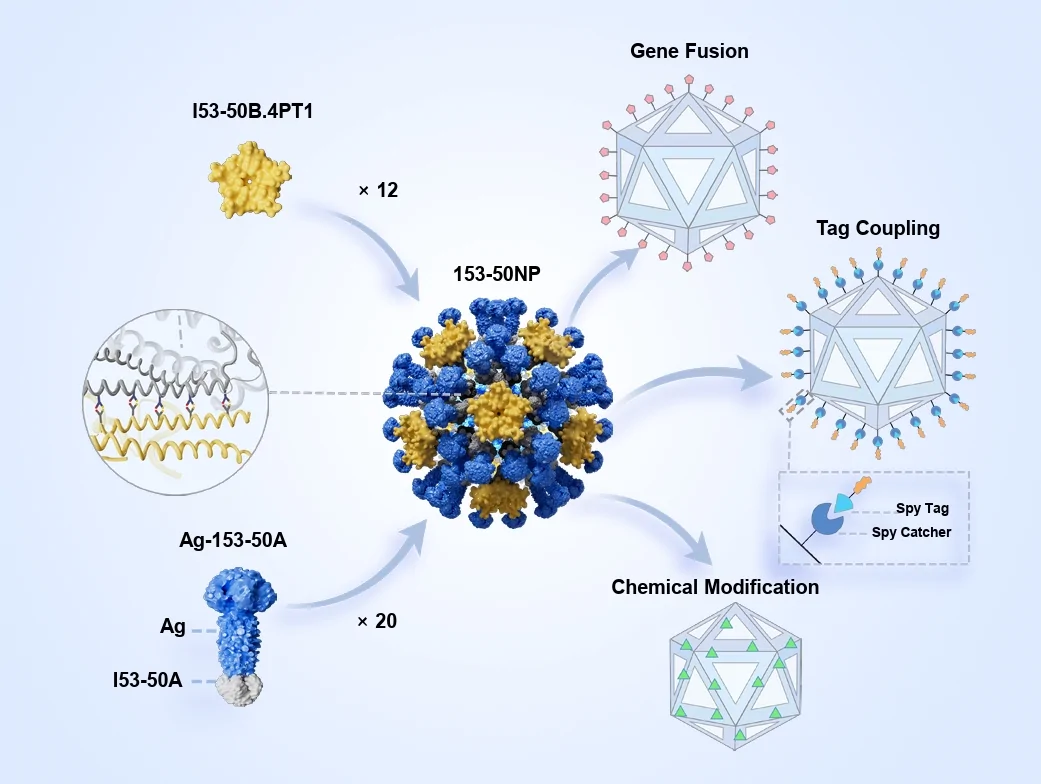
Schematic diagram of I53-50 Nanoparticles structure and functionalization.

**Table 1.** Comparison of functionalization strategies for I53-50 nanoparticle

Functionalization strategy	Key characteristics	Advantages	Limitations
Chemical modification	Targeting intrinsic active residues (preferentially N-terminal/C-terminal regions) of I53-50 subunits to conjugate functional groups or molecules via chemical reactions, thereby achieving protein functional modification and enhancement (Brouwer et al., 2019).	1. **Site-specificity:** Enables precise targeting of defined amino acid sites.	1. **Potential structural perturbation:** Chemical reactions may affect the native protein conformation and stability.
		2. **High flexibility:** Compatible with a wide range of chemical reagents and reactions.	2. **Risk of heterogeneity:** Conjugation reactions can yield heterogeneous products.
		3. **Overcomes spatial constraints:** Linker design allows fine-tuning of the distance between the antigen and the nanoparticle surface (Sletten and Bertozzi, 2009).	3. **Dependence on reactive residues:** Efficiency is limited by the accessibility of reactive amino acids (Sletten and Bertozzi, 2009; Stephanopoulos and Francis, 2011).
Tag coupling	Introducing specific tags into I53-50 subunits via genetic engineering or chemical methods, and achieving site-specific covalent coupling through tag-specific recognition for antigen display, purification, or targeting (Bruun et al., 2018).	1. **Stable covalent linkage:** Antigen is covalently and stably integrated into the assembly.	1. **Insert size limitation:** Large antigen inserts may impair subunit folding and self-assembly.
		2. **High-density & Uniform display:** Enables precise, ordered presentation of 60-240 antigen copies per particle.	2. **Design complexity:** Requires optimization of linkers (flexible/rigid) to balance antigen presentation and structural integrity.
		3. **Streamlined production:** Single-step expression and self-assembly simplify manufacturing (Zakeri et al., 2012).	3. **Long redesign cycles:** New antigens require de novo gene construction and validation (Veggiani et al., 2016).
Gene fusion	Fusing the coding gene of the target antigen with the modified I53-50 subunit gene via recombinant DNA technology, allowing the fusion protein to self-assemble into functional nanoparticles with antigen display (Marcandalli et al., 2019).	1. **Modularity & Versatility:** "Plug-and-play" platform for rapid interchange of different SpyTag-fused antigens.	1. **Requires tag engineering:** Both carrier and antigen require genetic modification, which may affect their native properties.
		2. **Site-specific & Efficient:** Forms irreversible isopeptide bonds under mild conditions, ensuring high coupling efficiency and homogeneity.	2. **Potential tag immunogenicity:** The tags themselves could elicit immune responses (typically low).
		3. **Enhanced stability:** Covalent linkage improves complex stability during storage and in vivo (Chen et al., 2013).	3. **Additional conjugation step:** Involves an in vitro coupling step compared to genetic fusion (Bird et al., 1988).

**Table 2.** Comparative analysis of I53-50 nanoparticles with other self-assembling protein platforms

Parameter / Feature	I53-50NP	VLP	Ferritin	Mi3
Subunit composition	Composed of 24 trimeric (I53-50A) and 12 pentameric (I53-50B) subunits assembling with strict T = 1 icosahedral symmetry (Bale et al., 2016)	Typically assembles from identical subunits via quasi-equivalent T = 3 or T = 4 icosahedral symmetry, leading to potential structural variability (Fuenmayor et al., 2017)	24 subunits self-assemble into a cage with octahedral (O) symmetry (Zhang et al., 2021)	Trimeric subunits assemble into 2D/3D lattices or finite particles with D3 (dihedral) symmetry (Eom et al., 2024)
Thermal stability (Tm)	**Exceptionally high.** Melting temperature (Tm) > 95°C (for variant I53-50A.1NT1) and remains intact after 1-h incubation at 70°C (Walls et al., 2020)	**Moderate to high.** Varies by source; e.g., HBcAg VLP Tm ~70–75°C, but may exhibit kinetic instability (Mohsen and Bachmann, 2022)	**High.** Human heavy-chain ferritin (HFH) Tm ~75°C, but stability varies across species and is pH-sensitive (Zhang et al., 2021)	**High.** Designed Mi3 protein exhibits Tm ~77°C (Liu et al., 2021)
Assembly mechanism	**Strictly controlled co-assembly.** A and B components must mix in a precise stoichiometric ratio to form uniform ~50 nm particles. Enables mosaic display of distinct antigens (Wargacki et al., 2021)	**Condition-dependent self-assembly.** Spontaneous assembly under specific conditions (pH, ionic strength) can lead to heterogeneity in size and morphology (Nooraei et al., 2021)	**pH-dependent assembly/Disassembly.** Assembles at neutral/alkaline pH and disassembles at acidic pH. Useful for cargo loading but may raise concerns about in vivo stability in certain microenvironments (Chen et al., 2022)	**Self-assembly** into extended arrays or finite particles. Achieving uniform, finite-sized nanoparticles requires precise design control (Wu et al., 2025)
Functionalization	**Highly flexible & Precise.** Supports genetic fusion (to N/C-termini or specific loops of A/B subunits), chemical conjugation, and tag coupling. Allow precise presentation of 60 or 120 antigen copies with controlled orientation (Wargacki et al., 2021)	**Flexible but potentially disruptive.** Common genetic fusion to N/C-termini or the major immunodominant (Mohsen and Bachmann, 2022) region (MIR). Insertions can interfere with assembly, and copy number and orientation control is less precise (Mohsen and Bachmann, 2022)	**Primarily N/C-terminal genetic fusion.** Due to octahedral symmetry, maximum valency is limited to 24 copies, resulting in lower antigen density (Munir et al., 2024)	**Primarily genetic fusion** to specific sites on the trimeric subunit. Its dihedral symmetry results in antigen geometry distinct from icosahedral presentation (Wu et al., 2025)

**Table 3.** Application of I53-50 nanoparticles in vaccine development

Name of the vaccine	Construction methods	Immunization effects	Type of connection	Assembly efficiency and conditions
RBD-I53-50NP (Kang et al., 2021)	ΔN1-SpyCatcher first binds to I53-50A1.1PT1 and I53-50B.4PT1, and then fuses with SpyTag attached to the C-terminal end of the SARS-CoV-2 RBD, resulting in ΔN1-SpyCatcher-I53-50NP	RBD-I53-50NP showed stronger affinity for the receptor ACE2 and the neutralizing antibody CB6, indicating good affinity for BCR and triggering higher titers of antibody	tag coupling	I53-50A1.1PT1 and I53-50B.4PT1 were incubated in vitro at 250 mM NaCl, 50 mM Tris-HCl (pH 8.0), containing 5% (v/v) glycerol, and purified by size exclusion chromatography (SEC). No free RBD antigen or residual I53-50 dimer subunits were detected, with high subunit assembly and antigen loading conversion rates.
SARS-CoV2 S-I53-50NP (Arunachalam et al., 2021; Kang et al., 2021)	SARS-CoV-2 S-I53-50NP formed by fusing the C-terminus of the pre-fused SARS-CoV-2 S protein to the N-terminus of the I53-50A variant of I53-50A.1NT1, followed by incubation with I53-50B.4PT1	Enhanced activation of SARS-CoV-2 S protein-specific B cells in vitro induces a strong NAb response	gene fusion	SARS-CoV-2 S-I53-50A.1NT1 and I53-50B.4PT1 were mixed in buffer at 4°C or room temperature for several hours under the following conditions: 50 mM Tris (pH 8), 150 mM NaCl, 100 mM L-arginine, and 5% (w/v) sucrose. Assembly efficiency is typically very high (> 90%), yielding monodisperse nanoparticles with highly uniform morphology.
RBD-8G/12G/16G-I53-50NP (Arunachalam et al., 2021)	SARS-CoV-2 RBD was genetically fused to the N-terminus of I53-50A via a linker of 8, 12, and 16 glycine and serine residues, and later fused to I53-50B.4PT1 to form RBD-I53-50NP	RBD-NP immunization induced a robust and long-lasting neutralizing antibody response, eliciting a strong antigen-specific CD4 T-cell response that skillfully neutralized the mutant strain	gene fusion	I53-50A and I53-50B4.PT1 were incubated at room temperature (25–30°C) for 1–2 h in a buffer containing 250 mM NaCl, 50 mM Tris-HCl pH 7.4–8.0, and 5% glycerol. The subunit assembly conversion rate approached 100% (size exclusion chromatography (SEC) showed no free subunit peaks), with a polydispersity index (PDI) < 0.1 (determined by dynamic light scattering (DLS)).
E2E1-I53-50NP (Sliepen et al., 2022)	The N-terminus of I53-50A1.NT1 is fused to the C-terminus of E2E1, which later fuses with I53-50B.4PT1 to form E2E1-I53-50NP	The number of neutralizing viruses in animals immunized with E2E1-NPs was significantly higher than that in animals receiving monoimmunization, and both the width and potency of Nab were significantly improved, with an increase in potency of up to 80-fold	gene fusion	E2E1-I53-50A and I53-50B.4PT1 were incubated overnight at 4°C in Tris-buffered saline (TBS, pH ~7.5) supplemented with 5% glycerol or L-arginine, achieving assembly efficiencies typically > 90%. Both SEC monomodal profiles and NS-EM images indicate the product consists of highly uniform protein nanoparticles, with no detectable free protein or aggregates.
p67C-I53-50NP (Lacasta et al., 2023)	ECF p67C is linked to the I53-50A N-terminus and the p67C-I53-50A fusion gene formed is fused to I53-50B.4PT1 to form p67c-I53-50NP	p67C-I53-50 immunized animals produced high p67C-specific IgG1 and IgG2 antibody titers as well as strong CD4+ T-cell responses, with the highest amount of IFN-γ secretion	gene fusion	p67C‑I53‑50A and I53-50B.4PT1 were incubated at room temperature (RT) for 30 min to 2 h in 50 mM Tris‑HCl, pH 8.0, 250–500 mM NaCl, supplemented with 50 mM glycine or 0.75% CHAPS, at room temperature (RT) for 30 min to 2 h. Size exclusion chromatography (SEC) revealed a single symmetric peak for the assembled nanoparticles, indicating a homogeneous product. Dynamic light scattering (DLS) showed a uniform particle size distribution consistent with the expected dimensions.
gB-I53-50-NP (Sun et al., 2023)	HCV viral gB gene fused to the N-terminus of I53-50A1 via C-terminal fusion, forming gB-I53-50A1 fused to I53-50B.4PT1 to form gB-I53-50NP	Induction of serum polyclonal antibodies by gB-I53-50NP vaccine in monkeys significantly protects humanized mice against EBV infection and lymphomagenesis, and the protective effect persists for a long period of time up to 10 weeks after immunization of monkeys	gene fusion	I53-50A1 and I53-50B4.PT1 can be efficiently assembled by incubating them for 1 h in assembly buffer (250 mM NaCl, 50 mM Tris pH 8.0, 5% glycerol).
SOSIP-I53-50NP (Brinkkemper et al., 2024)	HIV viral SOSIP Env trimer fused to I53-50A to form the SOSIP-I53-50A construct, which later fused to I53-50B.4PT1 to form SOSIP-I53-50NP	SOSIP-I53-50 NP in immunized animals, binds antibodies with reactive IgG titers up to 50–100 μg/ml, neutralizes viruses with titers ID50 > 100, Abs targets a wide range of neutralizing epitopes.	gene fusion	SOSIP-I53-50A and I53-50B.4PT1 were incubated overnight at 4°C in 25 mM Tris-HCl (pH 8.0), 500 mM NaCl, 5% glycerol at 4°C overnight. Dynamic light scattering analysis revealed the hydrodynamic radius of the assembled nanoparticles ranged from 246 to 292 Å with a low polydispersity index, indicating uniform and monodisperse particle size distribution.
DS-Cav1-I53-50 and Sc9-10-I53-50 (Hu et al., 2025)	The extracellular domain sequences of DS-Cav1 or Sc9-10 were fused with the I53-50A subunit gene to construct the DS-Cav1-I53-50A and Sc9-10-I53-50A fusion protein. The fusion protein was secreted and expressed in a stable Chinese hamster ovary (CHO) cell line, while the I53-50B subunit was expressed in *Escherichia coli* BL21 (DE3). The two subunits self-assembled in a 3:1 mass ratio to form DS-Cav1-I53-50 and Sc9-10-I53-50 nanoparticles.	In mouse models, total IgG titers, D25 competitive neutralization antibody titers, and live virus neutralization antibody titers against RSV A2 strain were elevated. Due to the antigenic properties of Sc9-10, Sc9-10-I53-50 exhibited slightly higher immunogenicity than DS-Cav1-I53-50.	gene fusion	DS-Cav1-I53-50A and Sc9-10-I53-50A were mixed with I53-50B in a buffer system of 50 mM Tris-HCl, 300 mM NaCl, 0.75% CHAPS, pH 7.4, and incubated at room temperature (RT) for 2 h. Purified by size exclusion chromatography (SEC), the particles exhibited uniform size and high assembly efficiency.
DS2-I53-50 (Jiang et al., 2025)	The DS2 antigen gene was directly fused with the I53-50A gene to construct the DS2-I53-50A fusion protein. The DS2-I53-50A fusion protein was transiently expressed in Expi293F mammalian cells, while the I53-50B subunit was induced in *Escherichia coli* BL21(DE3). Following separate purifications, the two subunits self-assembled in a 1:1 molar ratio to form stable DS2-I53-50 nanoparticles.	Following immunization of BALB/c mice, the induced DS2-specific IgG titer was 2.9 times that of free DS2, with the IgG1/IgG2a ratio decreasing to 2.6, indicating a Th1-biased response. This significantly promoted the proliferation of germinal center B cells (CD19⁺B220⁺CD95⁺GL-7⁺) and follicular helper T cells (CD4⁺CXCR5⁺PD-1⁺), activated dendritic cells and macrophages, and expanded the CD4⁺ central/ effector memory T cell populations.	gene fusion	Mix DS2-I53-50A with I53-50B in PBS (containing 5% glycerol) and incubate at 25°C for 3 h. Size exclusion chromatography shows that all particles assembled successfully, with the most uniform chromatographic peak observed for I53-50.
